# TRAJECTORIES OF HEALTH SYSTEM USE AND TRANSITIONS IN OLDER ADULTS WITH DEMENTIA

**DOI:** 10.1093/geroni/igz038.056

**Published:** 2019-11-08

**Authors:** Susan E Bronskill, Laura C Maclagan, Jennifer Walker, Jun Guan, Xuesong Wang, Ryan Ng, Marian J Vermeulen, Colleen J Maxwell

**Affiliations:** 1 Institute for Clinical Evaluative Sciences (ICES), Toronto, Ontario, Canada; 2 ICES, Toronto, Ontario, Canada; 3 Laurentian University, Sudbury, Ontario, Canada; 4 University of Waterloo, Waterloo, Ontario, Canada

## Abstract

Health systems strive to enable persons with Alzheimer’s and related dementias to remain at home to maximize their quality of life. There is limited evidence describing long-term trajectories of health system use by persons with dementia as they remain in the community over time. A cohort of 62,622 community-dwelling older adults was followed for seven years and matched to persons without dementia (controls) based on age, sex and comorbidities. Overall, persons with dementia were more likely than controls to use health services, particularly home care and hospitalizations with discharge delay; and were more likely to be admitted to a nursing home. As they remained in the community over time, persons with dementia used home care at an increasingly intensive rate. Our approach to examine trajectories of health system use among persons with dementia is of particular value to capacity planning initiatives to anticipate future health service needs of this population.

